# An Empirical Study of the Impact of Service Quality on Patient Satisfaction in Private Hospitals, Iran

**DOI:** 10.5539/gjhs.v7n1p1

**Published:** 2014-07-29

**Authors:** Ehsan Zarei, Abbas Daneshkohan, Behrouz Pouragha, Sima Marzban, Mohammad Arab

**Affiliations:** 1 Department of Public Health, School of Health, Shahid Beheshti University of Medical Sciences, Tehran, Iran; 2 Department of Public Health, School of Health, Albourz University of Medical Sciences, Karaj, Iran; 3 Department of Health Management & Economics, School of Public Health, Tehran University of Medical Sciences, Tehran, Iran

**Keywords:** service quality, Patient Satisfaction, patient-hospital relationship

## Abstract

**Objective:**

Perceived service quality is the most important predictor of patient satisfaction. The purpose of this study was to investigate the impact of the service quality on the overall satisfaction of patients in private hospitals of Tehran, Iran.

**Method:**

This cross-sectional study was conducted in the year 2010. The study’s sample consisted of 969 patients who were recruited from eight private general hospitals in Tehran, Iran using consecutive sampling. A questionnaire was used for data collection; contacting 21 items (17 items about service quality and 4 items about overall satisfaction) and its validity and reliability were confirmed. Data analysis was performed using t-test, ANOVA and multivariate regression.

**Result:**

this study found a strong relationship between service quality and patient satisfaction. About 45% of the variance in overall satisfaction was explained by four dimensions of perceived service quality. The cost of services, the quality of the process and the quality of interaction had the greatest effects on the overall satisfaction of patients, but not found a significant effect on the quality of the physical environment on patient satisfaction.

**Conclusions:**

Constructs related to costs, delivery of service and interpersonal aspect of care had the most positive impact on overall satisfaction of patients. Managers and owners of private hospitals should set reasonable prices compared to the quality of service. In terms of process quality, waiting time for visits, admissions, and surgeries must be declined and services provided at the fastest possible time. It should be emphasized to strengthen of interpersonal aspects of care and communication skills of care providers.

## 1. Introduction

Customer satisfaction as an important determinant of success and long-term survival in the health care industry has caught the providers’ attention in the present competitive conditions (Laohasirichaikul, Chaipoopirutana, & Combs, 2010). For the hospitals, satisfied patients are important because the patients’ greater satisfaction with the care would entail the patients’ more adherence of the doctor’s orders, more loyalty, positive word of mouth by the patient, reducing the number of the patient’s complaints, higher profitability, higher rates of the patient return and more patient referrals (Choi, Cho, S. Lee, H. Lee, & Kim, 2004; Dawn & Lee, 2004; Wu, 2011). For these reasons, the patient’s satisfaction evaluation has become a part of the strategic process of health care organizations. Measuring the patient satisfaction and recognition of its effective factors is important to the health care managers due to the impacts they make on the health and financial results of the health care organizations (Raposo, Alves, & Duarte, 2009).

Customer satisfaction is a general attitude that is formed based on the customer experience after the purchase of a product or consuming of a service that is manifested through an affective reaction in relation with the difference between what the customer expects and what he/she receives (Lai & Chen, 2011; Liu, Guo, & Lee, 2011). If the received services by the patient be weak and inconsistent with his/her expectations, he will then be dissatisfied. However if the received services conform to or beyond the patient’s expectations, this will result in his/her satisfaction (Laohasirichaikul et al., 2010). In other words, satisfaction reflects the degree to which a customer believes the usage of a service has caused positive feelings in him (Cronin, Brady, & Hult, 2000).

In the marketing literature, two customer satisfaction levels have been considered: Transactional-specific Satisfaction (transactional level) which means the customer’s evaluation and judgment concerning the purchase or consumption experience of a specific good or service (Deng, Lu, Wei, & Zhang, 2010) and the Overall Satisfaction (or cumulative satisfaction) meaning the customer’s evaluation and judgment concerning all purchases or consumption experiences of a specific goods or service (Wang, Lo, & Yang, 2004). Satisfaction at transactional level provides information as regards to the experience of an individual concerning a specific good or service, while overall satisfaction is a function of the benefits resulted from the consuming of a good or service. In other words, the overall satisfaction is comprised of all customer satisfaction or dissatisfaction in his/her transactions (Padma, Rajendran, & Prakash, 2010). At hospital service level, different services such as the admission, meals, nursing services, discharge, etc. each are considered as one transaction and the satisfaction resulting from these processes and services can be deemed as satisfaction at transaction level. Accordingly the satisfaction resulting from the whole care process and the services provided during the patient’s hospitalization period can be considered as overall satisfaction. According to marketing researchers’ arguments, since the overall satisfaction is a function of the sum of transactional satisfactions and reflects the customer’s feelings about the overall performance of an organization, so it would be more fundamental and useful in predicting the post-purchasing behavior of the customer (Wang et al., 2004).

The patient’s perception of the service quality plays an important role in achieving customer satisfaction and the causal relationship between the service quality and satisfaction has been an important topic of discussion in many relevant studies (Choi et al., 2004; Karatepe, 2011). Zeithaml, Berry and Parasuraman (1996) in their study of service quality consequences have pointed out that customer perception of the service quality is the most important predictor of the customer satisfaction. In practice, satisfaction and quality are often used interchangeably, but the consensus of researchers is that these are two distinct constructs, although highly correlated with each other (Padma et al., 2010). The quality judgments are relatively specific, while the satisfaction judgments are mainly general (Jen, Tu, & Lu, 2011). To achieve satisfaction, the patient should experience a service while the perceived service quality is not necessarily the result of an experience of a particular service (De Man, Gemmel, Vlerick, Van Rijk, & Dierckx, 2002). Also the quality of services is related to the cognitive judgments, while the customers’ satisfaction relates to the affective judgments (Choi et al., 2004; Lai & Chen, 2011). The differentiation between the service quality as a cognitive construct and the customers’ satisfaction as an affective construct suggests a causal relationship in which the service quality is a predictor for the patient satisfaction (Choi et al., 2004). H. Lee, Y. Lee and Yoo (2000) concludes that the customers are (dis) satisfied only when they have perceived and experienced the services; this shows that the service quality evaluation has priority over the customers’ satisfaction. Therefore the service quality is often seen as the customers’ satisfaction antecedent (Dabholkar, Shepherd, & Thorpe, 2000; Lei & Jolibert, 2012; Amin, Yahya, Ismayatim, Nasharuddin, & Kassim, 2013) and the notion that the service quality has a direct effect on satisfaction, has been widely accepted (Cronin & Taylor, 1992; C. M. Chen, S. H. Chen, & Lee, 2013).

The results of numerous studies conducted on the relationship between service quality and customer satisfaction show that higher quality of service will lead to higher satisfaction (Cronin et al., 2000; Bardy & Cronin, 2001; Lai, Griffin, & Babin, 2009). In the area of health services, the relationship between service quality and patient satisfaction has also been debated. The findings of the studies undertaken by Badri, Attia and Ustadi (2009) in the UAE and Yesilada and Direktor (2010) in Cyprus are indicative of positive effect of service quality on the patient’s satisfaction. In Iran, many studies have been done on patient satisfaction and quality of hospital service. For example, Arab et al. (2014) has conducted a study to design and validation of an instrument for measuring the inpatient satisfaction in hospital. They found seven dimensions for patient satisfaction: doctor-patient communication; nursing care; convenience; visitors; cleanliness; costs; and general satisfaction. In a systematic review by Amiresmaeil, Moosazadeh and Nekoei moghadam (2013), studies on patient satisfaction in Iran during 2001 to 2011 were reviewed. The study revealed that the rate of overall patient satisfaction was 70 %. But we did not find a study that has examined the relationship between service quality and patient satisfaction as two distinct constructs. Therefore, the aim of this study was the investing the impact of service quality dimensions on the overall satisfaction of the patients in private hospitals of Tehran, Iran.

## 2. Method

### 2.1 Study Design

This study was carried out in the year 2010 and the study target population was patients hospitalized in Tehran city private hospitals from which we selected 8 general hospitals for studying. The sample size in this study was 969 patients who were questioned on the day of discharging from hospital. Patients were selected consecutively and all the discharged patients were interviewed during the study period. The study aims were explained to the patients and they were assured of confidentiality of their personal information. As for the illiterate patients, a trained interviewer helped in filling out of the questionnaire.

### 2.2 Measurement Instrument

A researcher made questionnaire used for data gathering. The first part of the tool included 7 items related to the demographic characteristics of the patient. The second part included 17 items in the form of four quality dimensions: Physical environment quality with four items (Q1-Q4), process quality with 6 items (Q5-Q10) and interaction quality with 4 items (Q11-Q14). The items of this part were adapted of the SERVPERF questionnaire (Lei & Jolibert, 2012). In this study, we added up three items (Q15-Q17) to the questionnaire in relation to the costs to assess the effect of the cost on the quality perception. In several similar previous studies, the cost dimension was added to the other service quality dimensions (Rose, Uli, Abdul, & Ng, 2004; Anbori, Ghani, Yadva, Daher, & Su, 2010). The third part also included 4 items (SAT1-SAT4) regarding overall satisfaction of the patients. Items of this part were engineered on the basis of the studies undertaken by Lam, Shankar, Erramilli and Murthy (2004), Choi et al. (2004) and the Cronin et al. (2000).

### 2.3 Data Analysis

To evaluate the reliability of the questionnaire, the Cronbach’s alpha coefficient was calculated and the coefficients 0.934 and 0.942 were used for the “perceived quality” and the “overall satisfaction” indicative of stability and reliability, respectively. For the evaluation of the perception level of the service quality and overall satisfaction, the Likert scale (1 = totally disagree, 5 = totally agree) was used. The patient’s perceived service quality and overall satisfaction mean variable scores were obtained from the total items’ score divided into the number of items. Data analysis was performed using descriptive statistics, t-test, ANOVA and multivariate regression methods in SPSS.17 softaware.

### 3. Result

The average age of the patients was 48±16.9 of which 54.5% (528 people) were female and 45.5% (441 people) were male. About 7% of the patients were illiterate, 48% were from different higher education levels and 91% of the patients had medical insurance coverage. The average last of stay (ALS) in the hospital was 5.4±4.4 days and 33% of the patients had a previous stay in the current hospital. Also, 7.5% of the patients described their health status upon discharge from the hospital as “excellent”, 55% as “good” and 37.5% as “moderate” and “bad”.

The mean scores relevant to 17 items of service quality lay between 3.16 (Q16. Reasonable hospital service costs) and 4.38 (Q1. Well dressed and groomed staff). Among the four dimensions of service quality, the highest mean score (4.19) belonged to the physical environment quality (EQ) and the lowest mean score (3.39) related to the service costs’ dimension (Table 1). The total mean score of the patients’ perception of the hospital service quality was 3.91 ± 0.61 from 5. Also the mean scores pertaining to four items of overall satisfaction changed from 4.07 (SAT3. Making a wise decision for being hospitalized in this hospital) to 4.15 (SAT1. Overall satisfaction with the services provided by the hospital). The mean score of overall satisfaction also was 4.11±0.65 from 5 (Table 1).

**Table 1:** Mean and standard deviation scores of service quality and patient satisfaction items

Item/Dimension	Mean	±SD
**Environment Quality (EQ)**	4.19	0.59
Q1. Well dressed and groomed staff	4.38	0.53
Q2. Clean and comfortable environment of the hospital	4.32	0.58
Q3. Modern and up- to- date equipment	3.97	0.93
Q4. Visual appeal of physical facilities	4.14	0.75
**Process Quality (PQ)**	4.07	0.72
Q5. Telling when services will be performed	4.04	0.79
Q6. Prompt provision of medical and non-medical services	4.04	0.80
Q7. Willingness of staff to help patients	4.05	0.79
Q8. The availability of staff when needed	4.10	0.79
Q9. Creating a sense of trust in the patient	4.11	0.82
Q10. Conducting the services right at the first time	4.06	0.85
**Interaction Quality (IQ)**	3.74	0.79
Q11. Polite and friendly dealing with patients by staff	3.34	1.20
Q12. Attention to the patients’ beliefs and emotions	3.91	0.82
Q13. Having patients’ best interest at heart	3.85	0.85
Q14. Understanding the specific needs of patients	3.87	0.85
**Costs**	3.39	0.92
Q15. Costs versus quality of services	3.68	0.98
Q16. Reasonable hospital service costs	3.16	1.12
Q17. Valuable service versus paid costs	3.24	1.01
**Overall satisfaction**	4.11	0.65
SAT1. Overall satisfaction with the services provided by the hospital	4.15	0.67
SAT2. Satisfaction of selecting this hospital for hospitalization	4.12	0.69
SAT3. Making a wise decision for being hospitalized in this hospital	4.07	0.72
SAT4. Positive feeling about relationship with this hospital	4.09	0.72

Comparison of the overall satisfaction’s mean scores in terms of demographic variables of patient showed that having an insurance coverage (*t* = 2.53, *p* = 0.011), the hospital size (*t* = 2.09, *p* = 0.037) and health status upon discharge (*F* = 8.70, *p* < 0.000) affected the overall satisfaction of the patient, whereas variables like the age, length of stay, gender, education level and previous hospitalization in the current hospital had no effect. The patient’s overall satisfaction score of those with insurance coverage was higher than the patient’s without insurance coverage. The patients of large hospitals (over 150 beds) got higher overall satisfaction scores than those hospitalized in medium size hospitals (below 150 beds). Also the patients who described their health status at discharge as “excellent” had the highest and those who described their status as “bad” had the lowest overall satisfaction score.

To evaluate the relative significance of each of the service quality dimensions in prediction of the patient’s satisfaction, the linear regression analysis was performed. Based on the regression results, the *R^2^* value of this study model was 0.45 and therefore 45% of the variance of patient’s overall satisfaction is explained by the service quality. The regression coefficients show that the regression model was statistically significant and three independent variables of “service costs”, “process quality” and “interaction quality” were positively effective in the patient satisfaction, but the physical environment quality did not significantly affect the patient’s overall satisfaction (Table 2).

**Table 2:** Regression results: the impact of service quality on patient satisfaction

Service Quality dimensions	B	Beta	t-value	Sig.
Constant coefficient	1.56	-	13.845	< 0.001
Environment Quality	0.07	0.06	1.709	0.088
Process Quality	0.26	0.29	7.718	< 0.001
Interaction Quality	0.09	0.12	3.522	< 0.001
Cost	0.26	0.36	13.11	< 0.001

Adjusted R^2^ = 0. 450; F = 199.214, p < 0.001.

## 4. Discussion

The main aim of this study was investigating the service quality effect on the patient’s overall satisfaction in Tehran city private hospitals. The findings of this study showed that patients of Tehran’s private hospitals were highly satisfied with the quality of services they received. The four constructs used in this study explain 45% variance in the overall satisfaction of patients. *R^2^* values above 0.25 are indicative of great variance in the model (Cohen, 1988) and therefore it can be said that our hypothetical model features relatively good predictive power and is effective in explanation of the relationship between service quality and overall satisfaction from the patient’s perspective in the private hospitals which is consistent with the previous findings (Badri et al., 2009; Zamil, Areiqat, & Tailakh, 2012).

The patients without medical insurance coverage had a lower overall satisfaction score. In the previous studies also it has been determined that the evaluation of the patients with insurance coverage compared with the patients without such protection is more positive concerning the hospital’s services they received (Bakar, Akgün, & AlAssaf, 2008). The patient’s health status had also been influential on the patient’s overall satisfaction from the hospital and the patients who had evaluated their health status as good had higher overall satisfaction score. The previous studies’ findings confirm that the patient’s better physical and mental health status has a significant effect on their evaluation of the received services (Rahmqvist, 2001; Bacon & Mark, 2009). Also results of the studies undertaken by Badri et al. (2009) in the UAE and Tateke, Woldie and Ololo (2012) in Ethiopia showed that self-reported health status was positively correlated with the patient’s satisfaction. It seems like the more the patients feel recovered from their illness upon discharging from the hospital, they will have higher satisfaction from the hospital and its provided services. The patients of large hospitals had more overall satisfaction than those hospitalized in the medium and small sized hospitals. It appears that larger hospitals have more resources, facilities and workforce and can better serve the patients and meet their needs. In the previous studies also the hospital size has been reported as one of the variables effective in the patients’ satisfaction (Young, Meterko, & Desai, 2000).

The patients’ perception of the services’ costs had the greatest effect on their overall satisfaction, so that the more positive this perception was the patients felt more satisfied which is consistent with the previous study results (Anbori et al., 2010). Price of medical services is one of the most important determinants for many people in accessing and using such services. In the study conducted by Arab et al. (2014) the service costs were one of the determining factors in satisfaction of the Iranian hospital inpatients.

Process quality was the second effective factor in the overall satisfaction of patients. The findings of the studies carried out in Cyprus (Yesilada & Direktr, 2010), South Korea (Choi et al., 2004) and the UAE (Badri et al., 2009) show that the health services’ process quality and ease of access to such services had a significant effect on the satisfaction of the inpatient and outpatients. In the traditional approach of health care, the service quality was based on the quality of the care and not on the process of its delivery. Accordingly, it was assumed that if the patients received appropriate care and treatment, they will be satisfied (Choi, Lee, Kim, & Lee, 2005). But now the process of care provisioning for the patients, is just as important in establishing satisfaction in the patients as the technical quality of care. Senti and LeMire (2011) in their study reported that fast response to the patient’s request for assistance or relief of pain is the most important factor influencing the patient’s satisfaction. Fast service delivering was the second important factor in the patient satisfaction in the study conducted by Senic and Marinkovic (2013) in Serbia. One of the major problems affecting the process quality is the delays in the delivery process of the services in the hospital. If the patient assesses such delays unreasonable and unnecessary, this can lead to his/her dissatisfaction and anger (Duggirala, Rajendran, & Anantharaman, 2008).

Interaction quality was the third effective factor in the overall satisfaction. Previous studies’ results show that the patient-physician relationship and interpersonal aspects of the care are of the important and determining factors in the patient satisfaction (Keating et al., 2002; Raposo et al., 2009). Since the involvement of the service providers in the delivery of hospital services is inevitable, this finding can therefore be deemed as logical that patients assign more importance to the way the personnel interact with them. These results are definitely indicative of the fact that private hospitals and especially its personnel and physicians are to be encouraged and advised to establish strong interpersonal relationship with their patients.

The quality of the physical environment of service delivery had a small effect on overall satisfaction which is consistent with the results obtained from the previous studies. Similar studies undertaken in South Korea (Kim, Cho, Ahn, Goh, & Kim, 2008) and India (Rao, Peters, & Bandeen, 2006) determined that quality of infrastructure and hospital environment has no significant effect on the overall satisfaction of the patients. The physical environment quality among the service quality dimensions is a factor expected from the private hospitals and its inexistence will cause dissatisfaction of the patient; however attaining this factor has a small effect on the patient satisfaction (Kim et al., 2008).

Our study has limitations. First; this study has focused on the quantitative findings, however, in order to get better results, it should be improved using qualitative data. Evaluation of the services by the patient is a subjective process and using a quantitative tool like questionnaire cannot reflect all the patient’s judgments. Using qualitative methods, therefore besides the quantitative methods in the future studies could provide better understanding of the relationship between the service quality and the patient’s satisfaction. Second; results of this study have been obtained on the basis of study in Tehran city private hospitals, and since service provisioning by the public hospitals is by nature different from that of the private hospitals, it is recommended that other studies are carried out in the public hospitals in order to enhance the knowledge and information concerning the relationship between these two variables.

## 5. Conclusion

As expected, this study also found a strong relationship between service quality and patient satisfaction. But when service quality is studied as a multidimensional construct, provide invaluable tips for managers and decision-makers as well. Study of service quality as a multidimensional construct makes clear the effective areas of service quality in establishing patient satisfaction. Thus, managers can focus their quality improvement efforts on areas of service quality that have greater impact on patient satisfaction. The present study indicated that service’s costs, service delivery process and interaction with the patient had the most important positive effects on the overall satisfaction. For the hospital managers, this study emphasizes on the need for observing the tariffs and preserving high standards in the service provisioning process. The managers and owners of private hospitals must define rational prices in relation to the service quality. As regards to process quality, they must decrease the waitng time for the visits, hospitalization and surgery operations so that the services are deliverd as fast as possible. Also strengthening the interpersonal aspects of care and communication skills of doctors, nurses and staff should be emphasized.
